# Income in relation to obesity measures in an East German adult population: findings from the LIFE-Adult-Study

**DOI:** 10.1186/s12889-021-11302-w

**Published:** 2021-07-05

**Authors:** Cornelia Enzenbach, Bernd Kowall

**Affiliations:** 1 Institute for Medical Informatics, Statistics, and Epidemiology, University of Leipzig, Haertelstrasse 16-18, 04107 Leipzig, Germany; 2 LIFE - Leipzig Research Centre for Civilization Diseases, University of Leipzig, Philipp-Rosenthal-Strasse 27, 04103 Leipzig, Germany; 3 Institute for Community Medicine, Department SHIP-KEF, University Medicine Greifswald, Walter-Rathenau-Strasse 48, 17475 Greifswald, Germany; 4 Institute for Medical Informatics, Biometry, and Epidemiology, University Hospital Essen, Hufelandstrasse 55, 45147 Essen, Germany

**Keywords:** Obesity, Income, Socioeconomic status, Cross-sectional study, Germany

## Abstract

**Background:**

Obesity has been postulated to be a consequence of economic disadvantage. However, epidemiological studies failed to demonstrate a consistent link between income and body fat indicators. We examined income as a possible cause of obesity in an East German general population, focusing on appropriate representation of study variables, as well as on confounding and modification of the income-obesity association.

**Methods:**

We used data of 9599 participants in the baseline examination of the LIFE-Adult-Study, conducted in the city of Leipzig from 2011 to 2014. Body mass index (BMI) and waist circumference (WC) as obesity measures were based on standardised measurements, net equivalised income (NEI) on self-reports. We estimated adjusted means of BMI and WC within NEI categories representing the range from risk of poverty to affluence. We stratified the analyses by gender, age, and education.

**Results:**

A substantial part of the age-adjusted associations of income with obesity measures was attributable to other SES indicators. Adjusted for these variables, NEI was comparably associated with BMI and WC. Among women, BMI and WC decreased across NEI categories. The inverse associations tended to be stronger at non-working age (≥ 65 years) than at working age (< 65 years). Conversely, among working-age men, BMI and WC increased with increasing NEI. Among older men, risk of poverty was related to higher values of the obesity measures. The aforementioned associations were predominantly stronger in highly educated participants compared to those with medium/low education. The differences in mean BMI and WC between persons at risk of poverty and higher income groups were rather small, ranging from 1 to 2 kg/m^2^ for BMI and 2 to 4 cm for WC.

**Conclusions:**

Our investigation indicates an association between income and body fatness in an East German adult population that depends on the sociodemographic context of the people. However, it does not suggest that income disparities are a major driver of body fat accumulation in this population. Differential selection of study participants, error in the measurement of long-term income, and possibly reverse causality may have affected our conclusions.

**Supplementary Information:**

The online version contains supplementary material available at 10.1186/s12889-021-11302-w.

## Background

Obesity has become one of the most serious public health challenges of modern societies [1–4]. Excess body fat essentially results from a long-term positive energy balance, which in turn arises from an interaction between genetic predisposition and environmental factors [4, 5]. Among the latter, socioeconomic factors are particularly important because they can influence a variety of more proximal factors in the causal chain such as dietary intake, physical activity, and psychosocial characteristics [6]. Income is the indicator of socioeconomic status (SES) that most directly measures the material resources component [7]. Three main mechanisms through which income may affect body fat accumulation have been proposed: (1) Low-income groups preferably purchase low-cost food, which is typically characterised by high energy density, leading to chronic overconsumption of energy [8–12]. (2) Low income sorts people into deprived neighbourhoods with fewer opportunities for healthy diets and physical activity [8, 9, 11–13]. (3) Economic deprivation is related to psychosocial traits that favour increased energy intake in energy-rich environments, such as low levels of self-esteem, self-control, and social support, as well as social anxiety [9, 11, 12, 14, 15]. Against this background, obesity has been postulated to be “the toxic consequence of economic insecurity and a failing economic environment” [8].

In contrast to this hypothesis, income was frequently not related to obesity measures in adult populations of highly developed economies (in 45% and 61% of the reviewed studies in women and men, respectively) [16]. Observed associations were mainly inverse in women (in 88% of the studies), but more frequently positive in men (in 63% of the studies). In the European Union as a whole, obesity was found to be concentrated among the less affluent, although the inequality was low in magnitude [17]. It was again observed more consistently in women than in men. A small number of studies has investigated associations of income with obesity in the German general population [18–22]. Inverse associations were found in all of them. In line with the observations in other high-income countries, relations were seen more often in women (in all studies) than in men (in two of the five studies).

Despite numerous studies on the income-obesity association conducted so far, some methodological and content issues warrant a more thorough investigation:

Most studies relied only on body mass index (BMI), which is weight divided by height (kg/m^2^), as a measure of obesity. BMI is strongly correlated with whole-body fat mass [23], yet it is questioned that it adequately captures abdominal fat, which may be most relevant for the risk of cardiovascular and metabolic diseases [24]. Therefore, the need to consider other indicators in addition to BMI in the context of income-related inequalities in obesity has been emphasised [25, 26].

Usually household income is measured and adjusted for household size, resulting in the net equivalised income (NEI) [7]. For data analysis, quantiles based on the NEI distribution in the study sample are frequently computed. Alternatively, NEI can be categorised based on the distribution in the study region. Individuals in households with a NEI of less than 60% of the regional median NEI are commonly considered at risk of poverty, while those with more than 150% of the median NEI are classified as affluent [27–29]. Extreme income groups relative to the regional income distribution are not well characterised in terms of obesity by the available literature.

Other SES indicators, particularly education and occupation, are strongly related to both body size [16] and income [7]. The resulting confounding of the associations of income with obesity measures has not been thoroughly examined in the literature.

The relevance of income for obesity is likely to vary by population group. The direction of the relationship is known to differ by the economic development of a country [16], but also within countries of an economic region [17]. In East Germany, income inequality is a quite new phenomenon due to the egalitarian income policy of the former German Democratic Republic. Variation of the income-obesity association within Germany has rarely been studied, and an only slightly weaker relation in the East German than in the West German population was observed [18]. As outlined above, while an inverse association of income with body size is well established among women in western societies, the relation is less clear in men. Further, the meaning of current income may be most sensitive during the prime earning years, yet for older adults a less reliable indicator of their true SES [7]. In the few studies exploring this issue, income inequality in obesity varied inconsistently by age [17, 21]. Lastly, education may protect against the obesogenic effects of income disparities through knowledge acquisition and social capital [7]. However, epidemiological evidence for education being a modifier of the income-obesity association is scarce [30, 31].

Our investigation aimed at characterising income as a possible cause of obesity in an East German general population. We used cross-sectional data to examine: (1) Is income related to obesity measures in the adult population of the city of Leipzig? (2) What is the impact of other SES indicators on the observed associations? (3) Do sociodemographic factors modify the associations of income with body fat measures? We addressed existing research deficits by (a) focusing on an East German population, (b) considering not only BMI but additionally waist circumference (WC) as an indicator of the metabolically probably most relevant body fat distribution, (c) describing societally relevant income groups in terms of the distribution of obesity measures, (d) adjusting for confounding by other status indicators, namely education, occupation, and employment, and (e) evaluating the relevance of income for body fatness in as yet insufficiently characterised population groups: women versus (vs.) men, persons of working age vs. older persons, people with higher vs. lower educational level.

## Methods

### Study design and participants

We used data of the baseline examination of the LIFE-Adult-Study, a cohort study in the general population of the city of Leipzig [32]. From 1949 to 1990, Leipzig belonged to the German Democratic Republic. Today the city is located in the German federal state of Saxony. It had about 532,000 inhabitants of mostly central European descent in 2013 [33].

Participants in LIFE-Adult are mainly a random sample of Leipzig residents aged 40 to 79 years (y), which was drawn by the registration offices. In addition, a small sample of younger adults and volunteers was invited to study participation. In total, 10,000 participants had been planned as the LIFE-Adult population and were recruited from August 2011 to November 2014 (see [32] for more details on recruitment). In the present investigation, we included participants in the LIFE-Adult baseline examination who had valid values for all variables considered for the association analyses (*n* = 9599, Supplementary Figure S1).

### Variables and data assessment

The baseline examination was conducted in the LIFE-Adult study site involving trained study personnel.

The anthropometric measurements were carried out on participants wearing only underwear and being mainly in the fasting state. We calculated BMI from measurements of weight using an electronic scale (SECA 701, SECA GmbH & Co KG) and height using a stadiometer (SECA 240). WC was measured at the midpoint between the right lower ribcage and the right upper edge of the pelvic bone using an ergonomic measuring tape (SECA 201). Each anthropometric measure was taken once.

In a face-to-face interview, participants were asked to report their monthly net household income, the number of persons permanently living in the household, and the number of household members aged under 15. We calculated NEI by dividing net household income by the equivalised household size using the modified OECD equivalence scale [34]. We categorised NEI in order to account for non-linear associations with obesity measures and to depict societally relevant income disparities. We defined six categories according to common criteria of poverty and affluence research [27–29]. Persons in households with a NEI < 60% (category [cat] 1) and between 60% and < 80% (cat 2) of the regional median NEI were considered at risk of poverty, persons with a NEI of 80% to < 100% (cat 3) and 100% to < 150% (cat 4) of the median NEI constituted the middle income group, and those with a NEI of 150% to < 200% (cat 5) and a NEI ≥ 200% (cat 6) of the median NEI were regarded as affluent. We chose the population of the city of Leipzig in 2013 as the reference population. We used data of the German microcensus as an estimate of the income distribution in the Leipzig population (Statistical office of the Free State of Saxony, personal communication). NEI was comparably defined in LIFE-Adult and in the microcensus.

The variables considered potential modifiers and/or confounders of the associations of NEI with body fat measures are defined in Supplementary Table S1.

### Data analysis

#### Characterisation of the analysis population

We characterised the analysis population in terms of the distributions of BMI and WC using medians and interquartile ranges (IQR). We described the empirical associations between both body fat measures by means of Pearson correlation coefficients. We characterised the sample with regard to the distribution of NEI by calculating medians and IQR, as well as the frequencies of income levels according to the income distribution in the Leipzig population. We described sociodemographic characteristics of study participants by NEI category using relative and absolute frequencies (categorical variables) or median and IQR (metric variables).

#### Analysis of the associations of NEI with BMI and WC

We used multivariable linear models to analyse the associations between NEI and body fat measures. In a first model series, we examined the associations of interest considering gender and age as modifying factors. We estimated marginal (i.e., adjusted) means and their 95% confidence intervals (CI) of BMI and WC within NEI categories. We stratified the analyses by gender and age (< 65 y and ≥ 65 y, representing prime working age and non-working age in Germany) simultaneously. We considered a clear pattern of variation in the associations of interest across the strata an indication of modification [35]. We used three models to account for potential confounding of the relation between income and body fat measures. In model 1, we only adjusted for age as a biological confounder. In model 2, we additionally adjusted for education as the SES indicator that primarily represents the knowledge resources of an individual and probably affects health including body fatness mainly through this mechanism [7]. In model 3, we further adjusted for occupation and employment status (for persons aged under 65) as SES indicators that directly determine income and may impact body fatness to a relevant extent via this path [7]. We conducted several sensitivity analyses (only for BMI as the obesity measure) to investigate the consistency of our main results with those obtained with alternative model specifications. First, we used the log_e_-transformed BMI as the dependent variable. Second, we additionally adjusted for partner status and number of births (for females). Third, we adjusted for employment using a modified definition of this variable. Fourth, we examined employment instead of age as a modifier of the associations of interest.

In a second model series, we additionally considered education a modifier of the income-body fat marker association. For this purpose, we defined eight groups based on gender, age, and education (high vs. medium/low education). We had to summarise the six NEI categories into three categories (representing risk of poverty [cat 1], middle income [cat 2], and affluence [cat 3]) in order to ensure sufficient numbers of participants within each category. For each subgroup, we estimated means (95% CI) of BMI and WC within the three NEI categories, adjusting for age, education (metric variable), occupation, and employment.

The categorisation of NEI based on poverty and affluence criteria was accompanied by large differences in the income distribution between the strata defined by gender, age, and education. As one would expect, affluence according to the income distribution in the total (target) population concerned a relatively large proportion of the working population and those with high education, whereas it was a rather rare phenomenon in the non-working and lower educated strata. This heterogeneity in the income distribution might affect the comparison of the relationship of interest between the subgroups. In a third model series, we therefore calculated subgroup-specific quintiles of NEI in order to enhance comparability of income inequality, and also to reduce potential bias due to small person numbers in extreme categories. We regarded participants in quintile 1 as low-income, participants in quintile 5 as high-income, and participants in quintiles 2 to 4 as middle-income relative to the income position of participants with comparable sociodemographic context. With this alternative categorisation of exposure, we repeated the analyses described under the second model series.

We used SPSS (IBM SPSS Statistics), version 26, for our calculations.

## Results

### Characteristics of the analysis population

Women had lower medians (IQR) of BMI (26.1 [23.3–30.0] kg/m^2^) and WC (90.7 [82.6–100.1] cm) than men (BMI: 27.0 [24.7–30.0] kg/m^2^, WC: 100.0 [92.8–108.2] cm). BMI and WC were strongly correlated with each other. The strength of the correlation was similar for women and men, as well as for younger and older persons (Pearson correlation coefficient for women < 65 y: 0.91, women ≥65 y: 0.89, men < 65 y: 0.92, men ≥65 y: 0.90).

Female participants had a slightly lower median NEI (1467 [1100–2000] €) than male participants (1533 [1190–2021] €). The median NEI in Leipzig in 2013 had been estimated at 1265 €. The resulting six NEI categories representing the relative income position in the Leipzig population and their frequencies in LIFE-Adult are given in Table 1. For instance, 6.8% of female and 6.4% of male participants had less than 60% of the Leipzig median NEI (i.e., less than 759 €) and were thus considered at risk of poverty.

**Table 1:** Characteristics of study participants by category of net equivalised income

	Net equivalised income (euro)^a^					
	< 759	759 – < 1012	1012 – < 1265	1265 – < 1897.50	1897.50 – < 2530	≥ 2530
*Women*						
N (% of total sample)	342 (6.8)	693 (13.8)	705 (14.0)	1849 (36.8)	887 (17.7)	547 (10.9)
Age (years), median (IQR)	57.9 (47.8–63.7)	61.4 (49.8–68.5)	64.0 (51.6–70.7)	60.7 (48.2–70.0)	51.5 (45.5–58.9)	52.2 (46.1–57.7)
Living alone, % (n)	62.3 (213)	50.5 (350)	33.2 (234)	26.8 (495)	17.9 (159)	9.1 (50)
Educational level						
Low, % (n)	18.4 (63)	16.2 (112)	16.9 (119)	10.2 (189)	2.6 (23)	1.5 (8)
Medium, % (n)	58.8 (201)	62.5 (433)	60.1 (424)	55.5 (1027)	47.4 (420)	29.3 (160)
High, % (n)	22.8 (78)	21.4 (148)	23.0 (162)	34.2 (633)	50.1 (444)	69.3 (379)
Occupational status						
Low, % (n)	73.7 (252)	68.8 (477)	56.0 (395)	44.5 (823)	32.7 (290)	14.8 (81)
Medium, % (n)	19.6 (67)	26.4 (183)	38.3 (270)	44.0 (814)	48.9 (434)	46.1 (252)
High, % (n)	6.7 (23)	4.8 (33)	5.7 (40)	11.5 (212)	18.4 (163)	39.1 (214)
Employment						
Employed, % (n)	25.1 (86)	41.1 (285)	39.4 (278)	52.0 (962)	82.3 (730)	90.3 (494)
Unemployed, % (n)	39.8 (136)	9.5 (66)	2.7 (19)	1.8 (34)	0.8 (7)	1.1 (6)
Inactive, % (n)	35.1 (120)	49.4 (342)	57.9 (408)	46.1 (853)	16.9 (150)	8.6 (47)
*Men*						
N (% of total sample)	293 (6.4)	479 (10.5)	657 (14.4)	1645 (35.9)	817 (17.9)	685 (15)
Age (years), median (IQR)	55.9 (47.2–62.8)	63.5 (51.1–69.5)	66.2 (50.6–71.8)	63.5 (49.5–72.2)	51.7 (44.9–61.1)	53.5 (46.8–60.8)
Living alone, % (n)	60.4 (177)	30.5 (146)	18.4 (121)	13.7 (225)	16.3 (133)	12.1 (83)
Educational level						
Low, % (n)	18.1 (53)	19.4 (93)	14.9 (98)	8.4 (138)	2.0 (16)	1.5 (10)
Medium, % (n)	60.8 (178)	55.1 (264)	54.6 (359)	46.4 (763)	45.5 (372)	27.6 (189)
High, % (n)	21.2 (62)	25.5 (122)	30.4 (200)	45.2 (744)	52.5 (429)	70.9 (486)
Occupational status						
Low, % (n)	75.1 (220)	69.3 (332)	54.0 (355)	39.9 (657)	32.4 (265)	15.5 (106)
Medium, % (n)	19.5 (57)	26.1 (125)	39.0 (256)	48.0 (790)	52.1 (426)	50.9 (349)
High, % (n)	5.5 (16)	4.6 (22)	7.0 (46)	12.0 (198)	15.4 (126)	33.6 (230)
Employment						
Employed, % (n)	21.2 (62)	32.2 (154)	41.4 (272)	49.1 (808)	81.0 (662)	87.0 (596)
Unemployed, % (n)	52.6 (154)	13.2 (63)	2.4 (16)	2.2 (37)	1.0 (8)	0.7 (5)
Inactive, % (n)	26.3 (77)	54.7 (262)	56.2 (369)	48.6 (800)	18.0 (147)	12.3 (84)

*Abbreviation*: *IQR* interquartile range

^a^Categories of net equivalised income are based on the income distribution in the city of Leipzig in 2013. They represent < 60% (< 759 €), 60 – < 80% (759 – < 1012 €), 80 – < 100% (1012 – < 1265 €), 100 – < 150% (1265 – < 1897.50 €), 150 – < 200% (1897.50 – < 2530 €), and ≥ 200% (≥ 2530 €) of the median of the net equivalised incomes. See Table S1 for the definition of participants’ characteristics

Median age was lower in persons at risk of poverty and particularly in affluent participants compared to the middle-income groups (Table 1). Almost two third of the at-risk of poverty participants lived without a partner, this proportion markedly decreased across the income range. The percentage of highly educated persons substantially increased across the range of poverty to affluence (by factor 3.0 in women and 3.3 in men), as did the percentage of participants with high occupational status (by factor 5.8 in women and 6.1 in men). About 40% of female and 53% of male participants at risk of poverty were unemployed, while this figure was negligibly small in the middle- and high-income groups.

### Associations of NEI with BMI and WC: modification by gender and age

Adjusted for age only, NEI was inversely related to BMI among women, more so in younger than in older women (Table 2, model 1). Whereas mean BMI hardly differed across the six income categories among men aged under 65, mean BMI was higher in those at risk of poverty compared to the middle-income groups among older men.

**Table 2:** Estimated marginal means (95% CI) of BMI within NEI categories, stratified by gender and age

			NEI (euro)^a^					
			< 759	759 – < 1012	1012 – < 1265	1265 – < 1897.50	1897.50 – < 2530	≥ 2530
Women	< 65 years	N	271	437	378	1124	780	510
		NEI (euro)^b^	667 (592–704)	905 (833–1000)	1160 (1100–1200)	1533 (1385–1667)	2100 (2000–2333)	3000 (2667–3500)
		BMI (kg/m^2^)^c^, model 1	28.3 (27.7–29.0)	27.4 (26.9–27.9)	26.7 (26.2–27.3)	26.7 (26.4–27.0)	26.2 (25.9–26.6)	25.5 (25.0–25.9)
		BMI (kg/m^2^), model 2	28.1 (27.5–28.8)	27.3 (26.8–27.8)	26.6 (26.0–27.1)	26.6 (26.3–26.9)	26.3 (25.9–26.7)	25.8 (25.3–26.3)
		BMI (kg/m^2^), model 3	27.7 (27.1–28.4)	27.2 (26.7–27.8)	26.8 (26.2–27.4)	27.0 (26.6–27.4)	26.9 (26.4–27.4)	26.6 (26.0–27.2)
	≥ 65 years	N	71	256	327	725	107	37
		NEI (euro)	671 (600–716)	933 (867–1000)	1133 (1100–1200)	1467 (1333–1600)	2000 (2000–2235)	3000 (2667–3333)
		BMI (kg/m^2^), model 1	28.2 (27.1–29.4)	28.9 (28.3–29.5)	28.8 (28.2–29.3)	28.1 (27.8–28.5)	26.8 (25.9–27.8)	26.3 (24.7–27.9)
		BMI (kg/m^2^), model 2	28.1 (27.0–29.2)	28.7 (28.1–29.4)	28.7 (28.1–29.2)	28.2 (27.8–28.5)	27.2 (26.2–28.1)	26.7 (25.1–28.3)
		BMI (kg/m^2^), model 3	28.1 (27.0–29.2)	28.7 (28.1–29.3)	28.7 (28.1–29.2)	28.2 (27.8–28.5)	27.2 (26.2–28.2)	26.7 (25.1–28.4)
Men	< 65 years	N	246	261	307	882	674	588
		NEI (euro)	650 (548–700)	900 (833–1000)	1190 (1100–1200)	1559 (1389–1667)	2133 (2000–2333)	3167 (2750–4000)
		BMI (kg/m^2^), model 1	27.6 (27.1–28.1)	27.4 (26.9–27.9)	27.4 (27.0–27.9)	27.2 (27.0–27.5)	27.4 (27.1–27.8)	27.3 (27.0–27.6)
		BMI (kg/m^2^), model 2	27.4 (26.9–27.9)	27.3 (26.7–27.8)	27.4 (26.9–27.8)	27.2 (26.9–27.4)	27.5 (27.2–27.8)	27.5 (27.2–27.9)
		BMI (kg/m^2^), model 3	26.9 (26.3–27.4)	27.4 (26.8–27.9)	27.8 (27.2–28.3)	27.6 (27.2–28.0)	28.0 (27.6–28.4)	28.0 (27.6–28.5)
	≥ 65 years	N	47	218	350	763	143	97
		NEI (euro)	675 (650–733)	933 (867–1000)	1133 (1067–1200)	1467 (1333–1650)	2000 (2000–2250)	3000 (2667–4000)
		BMI (kg/m^2^), model 1	28.9 (27.7–30.0)	28.8 (28.2–29.3)	28.1 (27.7–28.5)	27.8 (27.5–28.1)	28.2 (27.6–28.8)	28.4 (27.6–29.1)
		BMI (kg/m^2^), model 2	28.7 (27.6–29.8)	28.6 (28.1–29.1)	28.0 (27.6–28.4)	27.9 (27.6–28.1)	28.3 (27.7–29.0)	28.6 (27.8–29.4)
		BMI (kg/m^2^), model 3	28.7 (27.6–29.9)	28.6 (28.1–29.2)	28.0 (27.6–28.5)	27.9 (27.6–28.1)	28.3 (27.7–29.0)	28.5 (27.7–29.3)

*Abbreviation*: *BMI* body mass index, *CI* confidence intervals, *NEI* net equivalised income

^a^Categories of NEI are based on the income distribution in the city of Leipzig in 2013. They represent < 60% (< 759 €), 60 – < 80% (759 – < 1012 €), 80 – < 100% (1012 – < 1265 €), 100 – < 150% (1265 – < 1897.50 €), 150 – < 200% (1897.50 – < 2530 €), and ≥ 200% (≥ 2530 €) of the NEI median

^b^Figures represent medians (interquartile ranges)

^c^Figures represent estimated marginal means (95% CI). Model 1: adjustment for age (metric variable), model 2: adjustment for age and education (metric variable), model 3: adjustment for age, education, occupation (metric variable), and employment status (employed, unemployed, inactive – only for participants < 65 years). See Table S1 for the definition of confounding variables

Additional adjustment for education attenuated the inverse income-BMI relation in women in both age groups (Table 2, model 2). It also weakened the BMI difference in older men, but hardly affected the BMI distribution across the income categories in younger men.

**Fig. 1:**
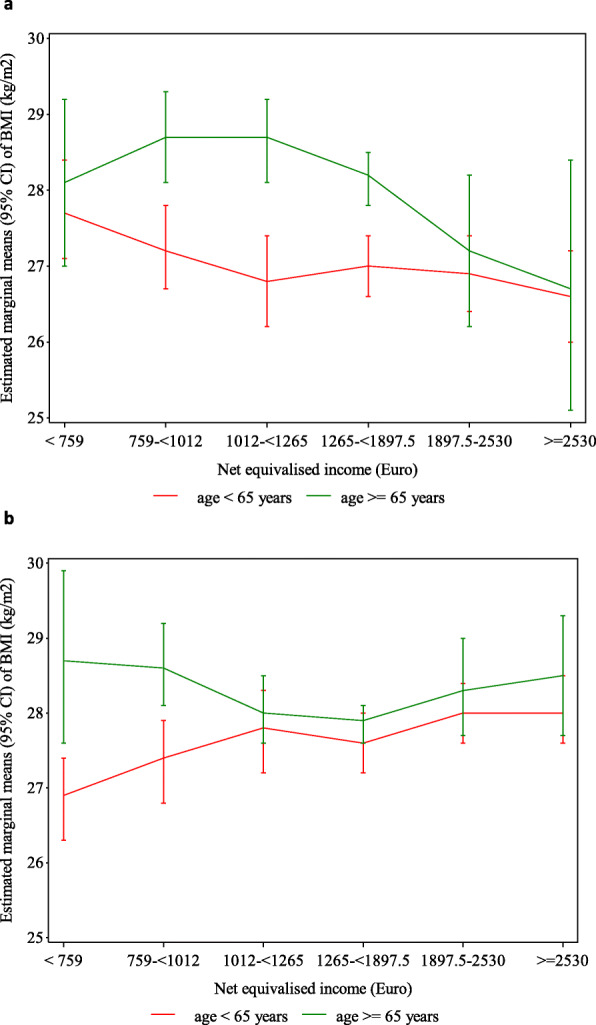
Estimated marginal means (95% CI) of BMI within NEI categories. **a** Women **b** Men. Categories of NEI are based on the income distribution in the city of Leipzig in 2013. They represent < 60% (< 759 €), 60 – < 80% (759 – < 1012 €), 80 – < 100% (1012 – < 1265 €), 100 – < 150% (1265 – < 1897.50 €), 150 – < 200% (1897.50 – < 2530 €), and ≥ 200% (≥ 2530 €) of the NEI median. The estimates are adjusted for age (metric variable), education (metric variable), occupation (metric variable), and employment status (employed, unemployed, inactive – only for participants < 65 years). See Table S1 for the definition of confounding variables. Abbreviation: BMI, body mass index, CI, confidence intervals

After further adjustment for occupation and employment, BMI was still inversely related to NEI in younger women, albeit markedly attenuated (Table 2, model 3, difference in mean BMI [95% CI], cat 1 vs. cat 6: 1.11 [0.19–2.03] kg/m^2^, see also Fig. 1a). In older women, mean BMI was not affected and higher in both the poverty and middle-income groups compared to the affluent groups (e.g., cat 2 vs. cat 6: 2.00 [0.25–3.75] kg/m^2^). In men aged under 65, NEI became positively related to BMI (cat 1 vs. cat 6: -1.19 [-1.99–-0.40] kg/m^2^, see also Fig. 1b). In older men, mean BMI remained higher in persons at risk of poverty compared to the middle-income groups (cat 1 vs. cat 4: 0.89 [-0.30–2.08] kg/m^2^).

The associations of NEI with WC within the four subgroups were comparable with those described for BMI as the outcome (Supplementary Table S2). The only difference referred to the age-adjusted association in younger men, which was invers for WC, whereas no association had been observed for BMI. Adjusted for age, education, occupation, and employment, the differences in mean WC between NEI categories were for women < 65 y, cat 1 vs. cat 6: 2.8 (0.6–5.0) cm; women ≥65 y, cat 2 vs. cat 6: 3.3 (-1.0–7.7) cm; men < 65 y, cat 1 vs. cat 6: -2.4 (-4.6–-0.2) cm; men ≥65 y, cat 1 vs. cat 4: 3.0 (-0.2–6.3) cm.

The results of sensitivity analyses were mostly consistent with those of the main analyses. Comparable associations were observed for the log_e_-transformed BMI as the dependent variable (data not shown). Likewise, additional adjustment for partner status and number of births yielded similar estimates (data not shown). Adjustment for employment applying a modified definition of this variable slightly strengthened the NEI-BMI association in younger men (cat 1 vs. cat 6: -1.36 [-2.16–-0.55] kg/m^2^), whereas the association in younger women was slightly weakened (cat 1 vs. cat 6: 0.85 [-0.10–1.80] kg/m^2^). Additional adjustment for employment did not affect the NEI-BMI relation in older participants, regardless of the definition of employment (data not shown). After stratification by gender and employment, NEI tended to be inversely related to BMI in both employed and retired women, although this mainly resulted from the BMI distribution in the extreme income groups with only few persons (Supplementary Table S3). In employed men, BMI strongly increased with increasing income, whereby particularly the small group of men at risk of poverty was characterised by a markedly lower mean BMI. In retired men, mean BMI did not differ relevantly across the income range.

### Associations of NEI with BMI and WC: modification by gender, age, and education

Among women aged under 65, NEI was inversely related to BMI in those with medium and low education (cat 1 vs. cat 3: 0.83 [0.07–1.56] kg/m^2^), whereas barely any association was seen in highly educated women (Table 3). In contrast, in older women and in men of both age groups, the NEI-BMI associations described above were observed in both educational groups (Table 3). In each case, these associations were stronger in highly educated participants (women ≥65 y, cat 1 vs. cat 3: 2.08 [0.29–3.87] kg/m^2^, men < 65 y, cat 1 vs. cat 3: -1.15 [-2.03–-0.27] kg/m^2^, men ≥65 y, cat 1 vs. cat 2: 1.16 [0.17–2.15] kg/m^2^) than in those with medium/low education (women ≥65 y, cat 1 vs. cat 3: 1.19 [-0.34–2.73] kg/m^2^, men < 65 y, cat 1 vs. cat 3: -0.59 [-1.33–0.14] kg/m^2^, men ≥65 y, cat 1 vs. cat 2: 0.39 [-0.30–1.07] kg/m^2^).

**Table 3:** Estimated marginal means (95% CI) of BMI within NEI categories, stratified by gender, age, and education

				NEI (euro)^a^		
				< 1012	1012 – < 1897.50	≥ 1897.50
Women	< 65 years	Medium/low education	N	531	1012	565
			NEI (euro)^b^	800 (700–933)	1400 (1250–1667)	2200 (2000–2667)
			BMI (kg/m^2^)^c^	28.1 (27.6–28.6)	27.8 (27.3–28.2)	27.3 (26.7–27.9)
		High education	N	177	490	725
			NEI (euro)	833 (680–950)	1500 (1333–1667)	2500 (2133–3000)
			BMI (kg/m^2^)	26.0 (25.3–26.8)	25.6 (25.0–26.2)	25.8 (25.1–26.4)
	≥ 65 years	Medium/low education	N	278	747	46
			NEI (euro)	900 (800–980)	1333 (1200–1467)	2000 (2000–2333)
			BMI (kg/m^2^)	28.7 (28.1–29.3)	28.6 (28.2–28.9)	27.5 (26.1–28.9)
		High education	N	49	305	98
			NEI (euro)	880 (733–987)	1467 (1280–1667)	2167 (2000–2667)
			BMI (kg/m^2^)	28.7 (27.2–30.1)	27.7 (27.1–28.3)	26.6 (25.5–27.6)
Men	< 65 years	Medium/low education	N	391	826	543
			NEI (euro)	750 (650–908)	1400 (1250–1667)	2333 (2000–2667)
			BMI (kg/m^2^)	27.5 (27.0–28.0)	27.9 (27.5–28.3)	28.1 (27.6–28.7)
		High education	N	116	363	719
			NEI (euro)	800 (668–913)	1467 (1250–1667)	2667 (2200–3333)
			BMI (kg/m^2^)	26.5 (25.8–27.3)	27.1 (26.5–27.7)	27.7 (27.1–28.2)
	≥ 65 years	Medium/low education	N	197	532	44
			NEI (euro)	910 (800–1000)	1267 (1133–1400)	2000 (2000–2517)
			BMI (kg/m^2^)	28.6 (28.0–29.2)	28.2 (27.9–28.6)	28.9 (27.7–30.1)
		High education	N	68	581	196
			NEI (euro)	933 (817–1000)	1467 (1333–1650)	2333 (2000–3000)
			BMI (kg/m^2^)	28.8 (27.9–29.8)	27.7 (27.4–28.0)	28.0 (27.5–28.6)

*Abbreviation*: *BMI* body mass index, *CI* confidence intervals, *NEI* net equivalised income

^a^Categories of NEI are based on the income distribution in the city of Leipzig in 2013. They represent < 80% (< 1012 €), 80 – < 150% (1012 – < 1897.50 €), and ≥ 150% (≥ 1897.50 €) of the NEI median

^b^Figures represent medians (interquartile ranges)

^c^Figures represent estimated marginal means (95% CI), adjusted for age (metric variable), education (metric variable), occupation (metric variable), and employment status (employed, unemployed, inactive – only for participants < 65 years). See Table S1 for the definition of confounding variables

The same pattern of heterogeneity between education levels was found for the association of NEI with WC (Supplementary Table S4).

### Associations of NEI with BMI and WC: quintiles of NEI

The distributions of BMI and WC across NEI quintiles showed similar patterns as across NEI categories based on poverty and affluence criteria (Table 4 and Supplementary Table S5). Merely the differences in mean BMI and WC between the income categories became smaller among older participants and larger among younger participants, which corresponded to changes in NEI variability (see Table 4 vs. Table 3). However, the heterogeneity in the associations of interest between the subgroups defined by gender, age, and education was still evident.

**Table 4:** Estimated marginal means (95% CI) of BMI within NEI quintiles, stratified by gender, age, and education

				NEI		
				Quintile 1	Quintile 2–4	Quintile 5
Women	< 65 years	Medium/low education	N	421	1177	510
			NEI (euro)^a^	762 (667–867)	1361 (1200–1667)	2250 (2000–2667)
			BMI (kg/m^2^)^b^	28.3 (27.7–28.8)	27.7 (27.2–28.1)	27.1 (26.5–27.8)
		High education	N	277	850	265
			NEI (euro)	980 (774–1130)	1944 (1600–2267)	3333 (3000–4000)
			BMI (kg/m^2^)	25.9 (25.2–26.5)	25.8 (25.2–26.4)	25.5 (24.7–26.3)
	≥ 65 years	Medium/low education	N	210	632	229
			NEI (euro)	852 (750–920)	1250 (1133–1333)	1667 (1533–1800)
			BMI (kg/m^2^)	28.7 (28.1–29.4)	28.7 (28.3–29.0)	28.1 (27.5–28.7)
		High education	N	90	268	94
			NEI (euro)	1000 (867–1100)	1533 (1333–1667)	2200 (2000–2667)
			BMI (kg/m^2^)	28.1 (27.0–29.1)	27.7 (27.1–28.3)	26.6 (25.6–27.7)
Men	< 65 years	Medium/low education	N	334	1055	371
			NEI (euro)	733 (607–840)	1500 (1250–1750)	2533 (2333–3000)
			BMI (kg/m^2^)	27.4 (26.9–27.9)	28.0 (27.6–28.4)	28.2 (27.6–28.9)
		High education	N	226	684	288
			NEI (euro)	1000 (800–1200)	2000 (1667–2400)	3517 (3200–4500)
			BMI (kg/m^2^)	26.5 (26.0–27.1)	27.6 (27.0–28.1)	27.5 (26.8–28.2)
	≥ 65 years	Medium/low education	N	134	498	141
			NEI (euro)	867 (750–930)	1200 (1067–1333)	1667 (1533–2000)
			BMI (kg/m^2^)	28.3 (27.6–29.0)	28.3 (27.9–28.6)	28.7 (28.1–29.4)
		High education	N	169	492	184
			NEI (euro)	1067 (973–1200)	1533 (1400–1667)	2467 (2000–3000)
			BMI (kg/m^2^)	28.4 (27.8–29.0)	27.6 (27.3–28.0)	28.0 (27.4–28.6)

*Abbreviation*: *BMI* body mass index, *CI* confidence intervals, *NEI* net equivalised income

^a^Figures represent medians (interquartile ranges)

^b^Figures represent estimated marginal means (95% CI), adjusted for age (metric variable), education (metric variable), occupation (metric variable), and employment status (employed, unemployed, inactive – only for participants < 65 years). See Table S1 for the definition of confounding variables

## Discussion

### Key results

Household income was related to both BMI and WC in an East German adult population. A substantial part of the age-adjusted associations of income with obesity measures was attributable to confounding by other SES indicators. Adjusted for these variables, the associations of interest varied by gender, age, and education.

Among women, BMI and WC decreased across categories representing the range from risk of poverty to affluence. The inverse associations tended to be more pronounced at non-working age than at working age. Conversely, among working-age men, BMI and WC increased with increasing income. Among older men, risk of poverty was related to higher values of the obesity measures. The aforementioned associations were mostly stronger among highly educated participants compared to those with medium/low education.

The differences in mean BMI and WC between persons at risk of poverty and higher income groups were generally small.

### Limitations

Although our investigation was based on a large data set, in our subgroups of interest, the extreme income categories representing risk of poverty and affluence partly comprised only few persons. As a result, the precision of our estimates was generally low as indicated by wide CI. In addition, biased estimates due to special characteristics of the few participants reporting extreme incomes are possible. Because of insufficient study size, we could also not stratify by working status directly but had to use prime working age as a surrogate in the main analyses, which might have masked important heterogeneity in the associations of interest.

Only about a third of the invited Leipzig residents participated in the LIFE-Adult baseline examination. As it is typical for studies on volunteers, this low participation was likely associated with a selection of individuals with higher SES and better health [36]. The associations between income and body fat measures might have been underestimated if the low-income persons in LIFE-Adult were less affected by overweight than their counterparts in the general population [37, 38].

Several sources of error in measurement of income are of concern. Income was assessed only at one point in time, although it can change considerably in the short term [7]. Further, income is a sensitive indicator with respect to participants’ willingness to disclose this information accurately [7]. It also may indeed be difficult to spontaneously provide accurate information on a complex quantity such as household income. Misreporting of the household’s composition might have additionally affected the level of equivalised income. Self-reported NEI should therefore be regarded only a rough estimate of the true long-term exposure. Misclassification of participants with respect to their actual NEI may have distorted our estimates of associations with obesity measures to an extent that we cannot judge. However, there are no indications that income was measured with greater error than in other studies in the general population [7, 39, 40].

Furthermore, it should be borne in mind that BMI and WC do not directly capture body fat, whose excess defines obesity. However, the accuracy of these simple indices was found to be sufficient for ranking participants’ body fatness and investigating its relations with health risks in large epidemiological studies [41].

Finally, we used cross-sectional data to reveal the relationship between income and body fatness in our population. Therefore, we cannot infer on the direction of the observed associations. We discuss the distinct interpretations following from this limitation in the next section.

### Interpretation of the results

The association between income and obesity found in social epidemiological research can be interpreted in two directions: (1) the causation hypothesis posits that low income causes obesity, whereas (2) the perspective of reverse causality views obesity as a cause of low income.

#### Reverse causality

A recent systematic review on the relative importance of causation and reverse causality in explaining the link between income and obesity found more consistent evidence for reverse causality, although the relationship is likely to be bidirectional [42]. The main argument for reverse causality is stigmatisation. Because of negative stereotypes, obese people face various weight penalties in the labour market in western societies [43]. This may particularly hold for women [44]. In contrast, in men, wages were found to be highest in the range from upper normal weight to obesity, probably reflecting the positive aura of physical strength in males [45].

Our data are consistent with this gender-specific link between income and obesity. However, reverse causality may be of minor importance in explaining the observations, at least among persons of non-working age. In this population group, income largely represents benefits from the public pension scheme, which depend mainly on wages over the life course. Their working life, in turn, these persons had largely spent in a socialist society where weight stigma is unlikely to have played an important role in employment. Nevertheless, we cannot rule out that reverse causality might have contributed to the inverse income-body size association in working-age women and the positive gradient in working-age men.

#### Causation hypothesis

Numerous studies aimed at evaluating income as a possible cause of obesity. In line with our observations, inverse associations were commonly found among women in high-income countries, including Germany [16–22, 46]. For men, the available data are contradictory: often no association was found [16, 17, 19, 21, 22], observed associations were frequently positive instead of negative [16, 46], yet in German general populations also negative [18, 20]. There is little evidence for gender differences in the relations of income to traits hypothesised to mediate an effect on body fatness. Studies among European and German adults showed that socioeconomic deprived groups consume less vegetables, fruit, and fibre than the better-off [47, 48]. Low-income persons were also less often engaged in sports activity than higher income groups in Germany [49]. However, neither the socioeconomic inequalities in dietary intakes nor in physical activity differed by gender. In contrast, a higher vulnerability to chronic stressors could explain why an inverse SES-obesity association is seen more consistently in women than in men [14, 50]. Moreover, gender differences in physical characteristics that are socially valued may underlie the observations in our study. For women, thinness represents the ideal of physical beauty and is materially more viable for those with higher SES, whereas for men, a larger body size is likely to be valued a symbol of physical dominance and prowess [16, 44].

Current income is considered a more reliable indicator of actual SES in the main earning years than at older ages [7]. Hence, the relation of income to body fatness is expected to be stronger at working age than at non-working age, a hypothesis that has been rarely investigated. Income inequality in obesity was reported to be both smaller and larger among women aged over 55 than in the middle-aged in European and German populations, respectively [17, 21]. In our women, the differences in body fat measures between the poor and the affluent tended to be larger among those of non-working age. Long-term income may have been captured more validly in retired women whose income mainly consists of stable pension benefits, which in turn reflect incomes over the life course [40]. Alternatively, the presence of employed women in the high-income groups among women aged over 65 may explain the unexpected observation. Still working women may be much more committed to the slimness ideal than non-working women. A sensitivity analysis did not point to heterogeneity in the income-BMI association between actually retired and employed women. In agreement with proposed mechanisms, the gradient in body fat measures across income categories was reversed and more pronounced among working-age men than among older men. Moreover, it was restricted to the employed when stratifying by employment directly.

We had hypothesised that education protects against the obesogenic effects of income inequality through cognitive skills and social capital. However, NEI was predominantly stronger related to body fat measures among highly educated participants, except in working-age women. The evidence for education as a modifier of the income-obesity relation is scarce. The inverse relation in South-Korean women was found to be stronger among the highly educated [30], which partly agrees with our findings. The stronger associations among highly educated persons may reflect a synergistic interaction between income and education with respect to the obesity-related pathways. On the other hand, self-reported income may be more valid for highly educated participants, although there is a lack of evidence on the relation between SES and the accuracy of income reporting [40]. In addition, the less educated population was probably less willing to participate in LIFE-Adult [36]. As a result, the observed dependencies of body fat measures from income may reflect the true situation less valid (are underestimated) in the less educated compared to the higher educated participants. Moreover, NEI varied more across income categories in highly educated persons, which may also have led to larger differences in obesity measures.

BMI and WC were strongly correlated in our population, indicating that both body fat surrogates measure approximately the same, which was later confirmed by their associations with income. Comparable correlations between BMI and WC were reported in the American general population [51]. In addition, both BMI and WC were highly correlated with direct measurements of body fat mass, body fat percentage, and subcutaneous adipose tissue, irrespective of age, gender, and ethnicity [23, 41]. In other social epidemiological studies, however, the association of income with obesity partly varied by the definition of the latter. Among British males, income was inversely related to WC but not to BMI [25]. Likewise, income was stronger related to WC than to BMI among Swedish women [26]. Confounding by other SES indicators may partly explain differences in the associations of income with WC compared to BMI. After adjustment for age only, we observed a decrease in WC with increasing income but no differences in BMI among working-age men, which agrees with the finding in British men. Consistent with our observation, education largely explained the income inequality in WC among the British men [25].

After adjustment for other SES indicators, the differences in mean BMI and WC between societally relevant income groups were rather small, ranging from 1 to 2 kg/m^2^ for BMI and 2 to 4 cm for WC. Hence, our data do not suggest that income disparities are a major driver of body fat accumulation in an East German population of middle and older age. Income inequality in obesity was low in magnitude in other European countries, too [17, 25]. Among German women, however, income turned out to be the strongest determinant of obesity relative to education and occupation [19]. Methodological limitations may have led to an underestimation of the true associations between income and body fat indices in the Leipzig population. On the other hand, even low income may be sufficient to meet the needs for healthy food, which is available at affordable prices in common supermarkets, at middle and older age in Germany. Also of importance, an obese phenotype develops in the long term and is probably significantly determined by early-life SES [44]. In our older population that had spent critical life periods in an egalitarian system, current income as a reflection of material conditions over the life course may not be a crucial factor for the distribution of obesity. Chronically overconsumption of calories has been primarily attributed to the psychosocial impact of living in a more hierarchical society [52].

## Conclusions

Our investigation indicates a relationship between income and body fatness in an East German adult population that varies by the sociodemographic context of the people. Both its strength and direction are subject to confounding by other SES indicators. The adjusted differences in obesity measures across categories representing the range from risk of poverty to affluence do not suggest that income disparities are a major driver of body fat accumulation in this population. Differential selection of study participants, error in the measurement of long-term income, and possibly reverse causality may have affected our conclusions. Thus, future research can improve on our study by quantifying the association of interest in a representative sample of the target population and by accounting for the dynamics of income over working life. In addition, longitudinal data can provide stronger evidence for the causation hypothesis.

## Supplementary Information

**Additional file 1: Figure S1.** Numbers of individuals at each stage of the LIFE-Adult-Study. **Table S1.** Definition of analysis variables: potential modifiers and/or confounders of the associations of net equivalised income with body fat measures. **Table S2.** Estimated marginal means (95% CI) of WC within NEI categories, stratified by gender and age. **Table S3.** Estimated marginal means (95% CI) of BMI within NEI categories, stratified by gender and employment status. **Table S4.** Estimated marginal means (95% CI) of WC within NEI categories, stratified by gender, age, and education. **Table S5.** Estimated marginal means (95% CI) of WC within NEI quintiles, stratified by gender, age, and education.

## Data Availability

The data analysed in the present study are available from the corresponding author on reasonable request.

## Abbreviations

BMI: Body mass index
cat: Category
IQR: Interquartile range
CI: Confidence interval
LIFE: Leipzig Research Centre for Civilization Diseases
NEI: Net equivalised income
SES: Socioeconomic status
WC: Waist circumference
y: Years
vs.: Versus
