# Supplementary material for: Income in relation to obesity measures in an East German adult population: findings from the LIFE-Adult-Study
Source: BMC Public Health. 2021 Jul 5;21:1313. doi: 10.1186/s12889-021-11302-w (PMC8256574; doi:10.1186/s12889-021-11302-w)
Supplement: Supplementary file 1 — Additional file 1: Figure S1. Numbers of individuals at each stage of the LIFE-Adult-Study. Table S1. Definition of analysis variables: potential modifiers and/or confounders of the associations of net equivalised income with body fat measures. Table S2. Estimated marginal means (95% CI) of WC within NEI categories, stratified by gender and age. Table S3. Estimated marginal means (95% CI) of BMI within NEI categories, stratified by gender and employment status. Table S4. Estimated marginal means (95% CI) of WC within NEI categories, stratified by gender, age, and education. Table S5. Estimated marginal means (95% CI) of WC within NEI quintiles, stratified by gender, age, and education. [file 12889_2021_11302_MOESM1_ESM.docx]

**Income in relation to obesity measures in an East German adult population: findings from the LIFE-Adult-Study**

Cornelia Enzenbach^1,2,3^, Bernd Kowall^4^

^1^Institute for Medical Informatics, Statistics, and Epidemiology, University of Leipzig, Haertelstrasse 16-18, 04107 Leipzig, Germany

^2^LIFE - Leipzig Research Centre for Civilization Diseases, University of Leipzig, Philipp-Rosenthal-Strasse 27, 04103 Leipzig, Germany

^3^Institute for Community Medicine, Department SHIP-KEF, University Medicine Greifswald, Walter-Rathenau-Strasse 48, 17475 Greifswald, Germany

^4^Institute for Medical Informatics, Biometry, and Epidemiology, University Hospital Essen, Hufelandstraße 55, 45147 Essen, Germany

Email addresses:

[cornelia.enzenbach@life.uni-leipzig.de](mailto:cornelia.enzenbach@life.uni-leipzig.de) (corresponding author)

bernd.kowall@uk-essen.de

Randomly sampled persons invited to LIFE-Adult

**32,195**

No contact possible:

Unknown postal address **826**

Deceased **95**

Invitation running **24**

Persons successfully contacted

**31,250**

Nonresponders

**12,196**

Refusals

**9,098**

Persons willing to participate

**468**

Participants

**9,488**

Volunteers

**512**

Missing data for:

BMI **36**

WC **47**

NEI **203**

Education **28**

Occupation **125**

Employment **84**

Data included in the analysis

**9,599**

Total recruited

**10,000**

Figure S1. Numbers of individuals at each stage of the LIFE-Adult-Study

*Invitation running* refers to those invitees who had been sent an invitation few weeks before the end of the recruitment and who did not respond within that time frame. *Persons willing to* *participate* are those invitees who had agreed to participate in LIFE-Adult but did not get an appointment because the targeted total number of participants had been achieved. *Refusals* are those invitees who actively declined to participate by means of a response form enclosed in the invitation letters or by phone. *Nonresponders* are those invitees who entirely ignored the invitation. Pregnancy and the inability to speak German were general exclusion criteria for participation in LIFE-Adult. Abbreviation: BMI, body mass index, NEI, net equivalised income, WC, waist circumference.

Table S1. Definition of analysis variables: potential modifiers and/or confounders of the associations of net equivalised income with body fat measures

| Variable  Role in the analyses | Data source  Details of assessment | Classification for analysis |
| --- | --- | --- |
|  |  |  |
| **Gender**  Modifier | Registration office | Female, male |
|  |  |  |
| **Age**  Modifier  Confounder | Registration office | As a potential modifier of the associations of interest, we dichotomised the metric age variable in *< 65 years* (representing prime working age) and *≥ 65 years* (representing non-working age), based on the statutory retirement age in Germany. |
|  |  |  |
| **Educational level**  Modifier  Confounder | Face-to-face interview  Participants were asked for their 1) highest general school leaving certificate and 2) professional qualifications. | We combined school and professional qualification based on the classification Comparative Analyses of Social Mobility in Industrial Nations (CASMIN) [S1]. We ranked the educational groups by assigning scores from 1 to 7, reflecting the average salaries earned in Germany by persons with the same qualifications, according to [S2]. As a potential modifier of the associations of interest, we dichotomised the interval-scaled variable in 1) *high education*: university entrance qualification/technical college qualification (CASMIN 2c-voc), university degree/technical college degree (CASMIN 3a, 3b) and 2) *low and medium education* (low education: no school *or* no professional qualification [CASMIN 1a, 1b, 1c, 2b], medium education: certificate of secondary education *and* training/apprenticeship/vocational school [CASMIN 2a], university entrance qualification/technical college qualification *and* no vocational qualification [CASMIN 2c-gen]). |
|  |  |  |
| **Occupational status**  Confounder | Face-to-face interview  Participants were asked for 1) their own current or former main employment activity, and 2) the current or former main activity of the partner (if existing). Answers had to be assigned to pre-defined occupational groups [S3]. | We operationalised occupational status as a household characteristic. We ranked occupational status of both the participant and the partner, based on the International Socio-Economic-Index of Occupational Status, and assigned scores from 1 to 7 according to [S2].We then assigned the higher of the two values to the household. For descriptive purposes, we categorised occupational status into tertiles. |
|  |  |  |
| **Employment status**  Confounder  Modifier (sensitivity analysis) | Face-to-face interview  Participants were asked 1) whether they were currently employed, and if not or less than 15 hours per week, 2) which group they belonged to out of eight status groups, including unemployed, pensioners, in vocational training. | We considered employment status a potential confounder of the associations of interest using two alternative definitions.  A. In the main analysis, we defined employment in line with the definition of the German microcensus [Statistical office of the Free State of Saxony, personal communication]. *Employed* are those persons who had been engaged in a gainful activity, even of a minor nature and not sufficient to earn a living, at the time of the interview. *Unemployed* are those persons who had not been gainfully employed, had declared themselves being unemployed and/or job-seeking, and had been able to start a new gainful activity within two weeks. *Inactive persons* are those persons who had not yet or no longer been gainfully employed.  B. In a sensitivity analysis, we modified definition A in that *Employment* is only given if a person had declared to be employed for at least 15 hours per week. We considered unemployed and inactive persons who had worked on an hourly basis as not employed and assigned them to their respective categories.  We considered employment status a potential modifier of the associations of interest in another sensitivity analysis. For this purpose, we distinguished between *Employed persons*, which are those persons who had been engaged in a gainful activity, regardless of its amount, at the time of the interview and *Retired persons*. We excluded unemployed and inactive persons other than pensioners from this analysis. |
|  |  |  |
| **Partner status**  Confounder (sensitivity analysis) | Face-to-face interview  Participants were asked for 1) their marital status and 2) whether they lived with a partner. | We dichotomised partner status in persons *living alone* and persons *living with a partner.* |
|  |  |  |
| **Number of births**  Confounder (sensitivity analysis) | Self-administered questionnaire  Female participants were asked how many children they had borne alive. | No classification was made. |

S1. Lampert T, Kroll LE. Die Messung des sozioökonomischen Status in sozialepidemiologischen Studien. In: Richter M, Hurrelmann K, editors. Gesundheitliche Ungleichheit: Grundlagen, Probleme, Perspektiven. 2nd ed, Wiesbaden: Verlag für Sozialwissenschaften; 2009. p. 309-34.

S2. Lampert T, Kroll LE, Müters S, Stolzenberg H. Messung des sozioökonomischen Status in der Studie „Gesundheit in Deutschland aktuell“ (GEDA). Bundesgesundheitsblatt Gesundheitsforschung Gesundheitsschutz 2013,56:131-43.

S3. Federal statistical office, editor. Demographische Standards: Eine gemeinsame Empfehlung des Arbeitskreises Deutscher Markt- und Sozialforschungsinstitute e. V. (ADM), der Arbeitsgemeinschaft Sozialwissenschaftlicher Institute e. V. (ASI) und des Statistischen Bundesamtes. 4th ed. Wiesbaden 2004.

Table S2. Estimated marginal means (95% CI) of WC within NEI categories, stratified by gender and age

|  |  |  | NEI (euro)^1^ | | | | | |
| --- | --- | --- | --- | --- | --- | --- | --- | --- |
|  |  |  | < 759 | 759 – < 1012 | 1012 – < 1265 | 1265 – < 1897.50 | 1897.50 – < 2530 | ≥ 2530 |
| Women | < 65 years | N | 271 | 437 | 378 | 1124 | 780 | 510 |
|  |  | NEI (euro)^2^ | 667 (592–704) | 905 (833–1000) | 1160 (1100–1200) | 1533 (1385–1667) | 2100 (2000–2333) | 3000 (2667–3500) |
|  |  | WC (cm)^3^, model 1 | 94.4 (93.0–95.9) | 92.6 (91.4–93.8) | 90.6 (89.4–91.9) | 90.6 (89.8–91.3) | 89.3 (88.4–90.2) | 87.9 (86.8–89.0) |
|  |  | WC (cm), model 2 | 94.0 (92.5–95.5) | 92.2 (91.0–93.4) | 90.3 (89.0–91.5) | 90.4 (89.7–91.2) | 89.5 (88.6–90.4) | 88.6 (87.5–89.8) |
|  |  | WC (cm), model 3 | 93.1 (91.6–94.6) | 92.0 (90.7–93.2) | 90.5 (89.2–91.9) | 91.1 (90.1–92.1) | 90.5 (89.4–91.7) | 90.3 (88.9–91.7) |
|  | ≥ 65 years | N | 71 | 256 | 327 | 725 | 107 | 37 |
|  |  | NEI (euro) | 671 (600–716) | 933 (867–1000) | 1133 (1100–1200) | 1467 (1333–1600) | 2000 (2000–2235) | 3000 (2667–3333) |
|  |  | WC (cm), model 1 | 95.8 (93.0–98.6) | 97.4 (95.9–98.9) | 97.1 (95.8–98.5) | 95.6 (94.7–96.5) | 92.5 (90.2–94.8) | 92.7 (88.8–96.6) |
|  |  | WC (cm), model 2 | 95.5 (92.7–98.3) | 97.1 (95.6–98.6) | 96.9 (95.6–98.3) | 95.7 (94.8–96.6) | 93.2 (90.9–95.6) | 93.6 (89.6–97.5) |
|  |  | WC (cm), model 3 | 95.4 (92.6–98.3) | 97.0 (95.5–98.5) | 96.9 (95.6–98.3) | 95.7 (94.8–96.6) | 93.3 (91.0–95.7) | 93.7 (89.7–97.7) |
| Men | < 65 years | N | 246 | 261 | 307 | 882 | 674 | 588 |
|  |  | NEI (euro) | 650 (548–700) | 900 (833–1000) | 1190 (1100–1200) | 1559 (1389–1667) | 2133 (2000–2333) | 3167 (2750–4000) |
|  |  | WC (cm), model 1 | 100.8 (99.3–102.2) | 100.0 (98.6–101.4) | 99.6 (98.3–100.9) | 99.2 (98.4–99.9) | 99.3 (98.4–100.2) | 98.9 (98.0–99.9) |
|  |  | WC (cm), model 2 | 100.2 (98.8–101.7) | 99.6 (98.2–101.0) | 99.4 (98.1–100.7) | 98.9 (98.1–99.7) | 99.5 (98.6–100.4) | 99.6 (98.6–100.6) |
|  |  | WC (cm), model 3 | 98.7 (97.1–100.2) | 99.9 (98.4–101.4) | 100.5 (99.1–102.0) | 100.1 (99.1–101.2) | 100.9 (99.7–102.1) | 101.0 (99.7–102.3) |
|  | ≥ 65 years | N | 47 | 218 | 350 | 763 | 143 | 97 |
|  |  | NEI (euro) | 675 (650–733) | 933 (867–1000) | 1133 (1067–1200) | 1467 (1333–1650) | 2000 (2000–2250) | 3000 (2667–4000) |
|  |  | WC (cm), model 1 | 106.7 (103.6–109.9) | 106.2 (104.8–107.7) | 103.9 (102.8–105.1) | 103.2 (102.4–104.0) | 104.5 (102.7–106.3) | 104.8 (102.6–107.0) |
|  |  | WC (cm), model 2 | 106.3 (103.1–109.4) | 105.8 (104.3–107.2) | 103.6 (102.4–104.8) | 103.4 (102.6–104.2) | 105.0 (103.1–106.8) | 105.5 (103.2–107.7) |
|  |  | WC (cm), model 3 | 106.4 (103.2–109.6) | 105.8 (104.3–107.3) | 103.6 (102.5–104.8) | 103.4 (102.6–104.2) | 104.9 (103.1–106.7) | 105.3 (103.0–107.6) |

^1^Categories of NEI are based on the income distribution in the city of Leipzig in 2013. They represent < 60% (< 759 €), 60 – < 80% (759 – < 1012 €), 80 – < 100% (1012 – < 1265 €), 100 – < 150% (1265 – < 1897.50 €), 150 – < 200 % (1897.50 – < 2530 €), and ≥ 200% (≥ 2530 €) of the NEI median. ^2^Figures represent medians (interquartile ranges). ^3^Figures represent estimated marginal means (95% CI). Model 1: adjustment for age (metric variable), model 2: adjustment for age and education (metric variable), model 3: adjustment for age, education, occupation (metric variable), and employment status (employed, unemployed, inactive – only for participants < 65 years). See Table S1 for the definition of confounding variables. Abbreviation: CI, confidence intervals, NEI, net equivalised income, WC, waist circumference.

Table S3. Estimated marginal means (95% CI) of BMI within NEI categories, stratified by gender and employment status

|  |  |  | NEI (euro)^1^ | | | | | |
| --- | --- | --- | --- | --- | --- | --- | --- | --- |
|  |  |  | < 759 | 759 – < 1012 | 1012 – < 1265 | 1265 – < 1897.50 | 1897.50 – < 2530 | ≥ 2530 |
| Women | Employed | N | 61 | 260 | 269 | 941 | 721 | 490 |
|  |  | NEI (euro)^2^ | 667 (600–733) | 933 (850–1000) | 1179 (1100–1200) | 1563 (1400–1667) | 2100 (2000–2333) | 3000 (2667–3500) |
|  |  | BMI (kg/m^2^)^3^ | 26.9 (25.6–28.2) | 26.0 (25.4–26.6) | 26.4 (25.7–27.0) | 26.3 (25.9–26.6) | 26.2 (25.8–26.5) | 25.8 (25.3–26.2) |
|  | Retired | N | 98 | 325 | 397 | 826 | 141 | 41 |
|  |  | NEI (euro) | 700 (620–733) | 933 (867–1000) | 1133 (1100–1200) | 1467 (1333–1600) | 2000 (2000–2200) | 2833 (2667–3333) |
|  |  | BMI (kg/m^2^) | 28.4 (27.4–29.4) | 28.9 (28.3–29.5) | 28.5 (28.0–29.0) | 28.4 (28.0–28.7) | 27.6 (26.8–28.5) | 28.0 (26.4–29.6) |
| Men | Employed | N | 51 | 145 | 271 | 806 | 658 | 595 |
|  |  | NEI (euro) | 600 (500–700) | 944 (867–1000) | 1200 (1100–1200) | 1590 (1400–1700) | 2133 (2000–2333) | 3250 (2778–4000) |
|  |  | BMI (kg/m^2^) | 24.9 (23.8–26.0) | 26.7 (26.0–27.3) | 26.9 (26.4–27.4) | 27.0 (26.7–27.2) | 27.5 (27.2–27.8) | 27.7 (27.3–28.0) |
|  | Retired | N | 68 | 251 | 366 | 793 | 145 | 82 |
|  |  | NEI (euro) | 699 (638–733) | 933 (867–1000) | 1133 (1067–1200) | 1467 (1333–1640) | 2000 (2000–2250) | 2667 (2667–3333) |
|  |  | BMI (kg/m^2^) | 28.3 (27.3–29.3) | 28.5 (28.0–29.0) | 28.2 (27.8–28.6) | 27.9 (27.7–28.2) | 28.3 (27.6–28.9) | 27.8 (27.0–28.7) |

^1^Categories of NEI are based on the income distribution in the city of Leipzig in 2013. They represent < 60% (< 759 €), 60 – < 80% (759 – < 1012 €), 80 – < 100% (1012 – < 1265 €), 100 – < 150% (1265 – < 1897.50 €), 150 – < 200 % (1897.50 – < 2530 €), and ≥ 200% (≥ 2530 €) of the NEI median. ^2^Figures represent medians (interquartile ranges). ^3^Figures represent estimated marginal means (95% CI), adjusted for age (metric variable), education (metric variable), and occupation (metric variable). See Table S1 for the definition of confounding variables. Abbreviation: BMI, body mass index, CI, confidence intervals, NEI, net equivalised income.

Table S4. Estimated marginal means (95% CI) of WC within NEI categories, stratified by gender, age, and education

|  |  |  |  | NEI (euro)^1^ | | |
| --- | --- | --- | --- | --- | --- | --- |
|  |  |  |  | < 1012 | 1012 – < 1897.50 | ≥ 1897.50 |
| Women | < 65 years | Medium/low education | N | 531 | 1012 | 565 |
|  |  |  | NEI (euro)^2^ | 800 (700–933) | 1400 (1250–1667) | 2200 (2000–2667) |
|  |  |  | WC (cm)^3^ | 94.2 (93.0–95.4) | 92.7 (91.6–93.8) | 91.3 (89.9–92.8) |
|  |  | High education | N | 177 | 490 | 725 |
|  |  |  | NEI (euro) | 833 (680–950) | 1500 (1333–1667) | 2500 (2133–3000) |
|  |  |  | WC (cm) | 88.5 (86.7–90.3) | 88.2 (86.7–89.6) | 88.4 (86.9–89.9) |
|  | ≥ 65 years | Medium/low education | N | 278 | 747 | 46 |
|  |  |  | NEI (euro) | 900 (800–980) | 1333 (1200–1467) | 2000 (2000–2333) |
|  |  |  | WC (cm) | 97.0 (95.6–98.5) | 96.5 (95.7–97.4) | 94.3 (90.8–97.8) |
|  |  | High education | N | 49 | 305 | 98 |
|  |  |  | NEI (euro) | 880 (733–987) | 1467 (1280–1667) | 2167 (2000–2667) |
|  |  |  | WC (cm) | 96.2 (92.7–99.7) | 94.9 (93.5–96.3) | 92.4 (89.9–94.9) |
| Men | < 65 years | Medium/low education | N | 391 | 826 | 543 |
|  |  |  | NEI (euro) | 750 (650–908) | 1400 (1250–1667) | 2333 (2000–2667) |
|  |  |  | WC (cm) | 100.4 (99.2–101.7) | 101.0 (99.9–102.2) | 101.4 (99.9–102.9) |
|  |  | High education | N | 116 | 363 | 719 |
|  |  |  | NEI (euro) | 800 (668–913) | 1467 (1250–1667) | 2667 (2200–3333) |
|  |  |  | WC (cm) | 97.6 (95.5–99.8) | 98.6 (97.0–100.3) | 99.7 (98.1–101.3) |
|  | ≥ 65 years | Medium/low education | N | 197 | 532 | 44 |
|  |  |  | NEI (euro) | 910 (800–1000) | 1267 (1133–1400) | 2000 (2000–2517) |
|  |  |  | WC (cm) | 106.0 (104.4–107.6) | 104.6 (103.6–105.5) | 105.7 (102.4–109.0) |
|  |  | High education | N | 68 | 581 | 196 |
|  |  |  | NEI (euro) | 933 (817–1000) | 1467 (1333–1650) | 2333 (2000–3000) |
|  |  |  | WC (cm) | 106.5 (104.0–109.2) | 102.6 (101.7–103.5) | 104.0 (102.4–105.6) |

^1^Categories of NEI are based on the income distribution in the city of Leipzig in 2013. They represent < 80% (< 1012 €), 80 – < 150% (1012 – < 1897.50 €), and ≥ 150% (≥ 1897.50 €) of the NEI median. ^2^Figures represent medians (interquartile ranges). ^3^Figures represent estimated marginal means (95% CI), adjusted for age (metric variable), education (metric variable), occupation (metric variable), and employment status (employed, unemployed, inactive – only for participants < 65 years). See Table S1 for the definition of confounding variables. Abbreviation: CI, confidence intervals, NEI, net equivalised income, WC, waist circumference.

Table S5. Estimated marginal means (95% CI) of WC within NEI quintiles, stratified by gender, age, and education

|  |  |  |  | NEI | | |
| --- | --- | --- | --- | --- | --- | --- |
|  |  |  |  | Quintile 1 | Quintile 2–4 | Quintile 5 |
| Women | < 65 years | Medium/low education | N | 421 | 1177 | 510 |
|  |  |  | NEI (euro)^1^ | 762 (667–867) | 1361 (1200–1667) | 2250 (2000–2667) |
|  |  |  | WC (cm)^2^ | 94.8 (93.5–96.1) | 92.4 (91.3–93.4) | 91.0 (89.5–92.5) |
|  |  | High education | N | 277 | 850 | 265 |
|  |  |  | NEI (euro) | 980 (774–1130) | 1944 (1600–2267) | 3333 (3000–4000) |
|  |  |  | WC (cm) | 88.2 (86.7–89.8) | 88.5 (87.2–89.9) | 87.8 (85.9–89.7) |
|  | ≥ 65 years | Medium/low education | N | 210 | 632 | 229 |
|  |  |  | NEI (euro) | 852 (750–920) | 1250 (1133–1333) | 1667 (1533–1800) |
|  |  |  | WC (cm) | 97.2 (95.5–98.8) | 96.7 (95.7–97.6) | 95.8 (94.2–97.4) |
|  |  | High education | N | 90 | 268 | 94 |
|  |  |  | NEI (euro) | 1000 (867–1100) | 1533 (1333–1667) | 2200 (2000–2667) |
|  |  |  | WC (cm) | 95.0 (92.4–97.6) | 94.9 (93.5–96.4) | 92.8 (90.2–95.3) |
| Men | < 65 years | Medium/low education | N | 334 | 1055 | 371 |
|  |  |  | NEI (euro) | 733 (607–840) | 1500 (1250–1750) | 2533 (2333–3000) |
|  |  |  | WC (cm) | 100.1 (98.8–101.5) | 101.1 (100.0–102.3) | 101.8 (100.2–103.5) |
|  |  | High education | N | 226 | 684 | 288 |
|  |  |  | NEI (euro) | 1000 (800–1200) | 2000 (1667–2400) | 3517 (3200–4500) |
|  |  |  | WC (cm) | 97.5 (95.9–99.2) | 99.7 (98.2–101.2) | 99.2 (97.3–101.1) |
|  | ≥ 65 years | Medium/low education | N | 134 | 498 | 141 |
|  |  |  | NEI (euro) | 867 (750–930) | 1200 (1067–1333) | 1667 (1533–2000) |
|  |  |  | WC (cm) | 105.3 (103.4–107.3) | 104.7 (103.7–105.7) | 105.7 (103.8–107.5) |
|  |  | High education | N | 169 | 492 | 184 |
|  |  |  | NEI (euro) | 1067 (973–1200) | 1533 (1400–1667) | 2467 (2000–3000) |
|  |  |  | WC (cm) | 104.5 (102.8–106.2) | 102.6 (101.6–103.6) | 104.0 (102.3–105.6) |

^1^Figures represent medians (interquartile ranges). ^2^Figures represent estimated marginal means (95% CI), adjusted for age (metric variable), education (metric variable), occupation (metric variable), and employment status (employed, unemployed, inactive – only for participants < 65 years). See Table S1 for the definition of confounding variables. Abbreviation: CI, confidence intervals, NEI, net equivalised income, WC, waist circumference.
